# Unsteady Aerodynamics in Bio-Inspired Flapping Wings for Low-Density Environments

**DOI:** 10.3390/biomimetics11060398

**Published:** 2026-06-05

**Authors:** Emilia Georgiana Prisăcariu, Oana Dumitrescu, Mihail Sima, Vlad Aparece-Scutariu, Sergiu Strătilă, Raluca Andreea Roșu, Cleopatra Cuciumita, Iulian Vlăducă, Silvia Bica

**Affiliations:** The Romanian Research and Development Institute for Gas Turbines COMOTI, 061126 Bucharest, Romania; emilia.prisacariu@comoti.ro (E.G.P.); mihail.sima@comoti.ro (M.S.); vlad.aparece@comoti.ro (V.A.-S.); sergiu.stratila@comoti.ro (S.S.); raluca.rosu@comoti.ro (R.A.R.); cleopatra.cuciumita@comoti.ro (C.C.); iulian.vladuca@comoti.ro (I.V.); silvia.bica@comoti.ro (S.B.)

**Keywords:** flapping flight, aeroelasticity, unsteady aerodynamics, low Reynolds number

## Abstract

Flapping-wing flight offers a promising solution for aerial mobility in low-density environments such as the Martian atmosphere, where conventional rotorcraft faces significant performance constraints. However, the coupled aerodynamic and structural mechanisms governing lift generation at low Reynolds numbers remain insufficiently understood. This study investigates the aeroelastic and unsteady aerodynamic behaviour of a bio-inspired flapping wing using an integrated experimental–numerical framework. High-speed imaging is employed to extract representative wing kinematics, including flapping frequency, stroke amplitude, and rotational motion. A geometrically scaled wing model is developed based on Reynolds number similitude and analysed using finite element methods to characterise its dynamic response. Aeroelastic behaviour is evaluated through modal transient simulations, while aerodynamic performance is assessed using both vortex-lattice modelling and computational fluid dynamics. The results show strong coupling between bending and torsional modes, with the structural response highly dependent on excitation frequency relative to the natural modes. Near-resonant conditions lead to amplified deformation and distinct phase relationships, while aerodynamic simulations reveal vortex-dominated lift generation. These findings provide a physics-based framework for the design and analysis of flapping-wing systems operating in low-Reynolds-number and low-density flight regimes.

## 1. Introduction

Aerial mobility has the potential to significantly enhance planetary exploration by enabling rapid terrain reconnaissance, atmospheric measurements, and access to locations unreachable by ground-based rovers. However, flight in the Martian atmosphere presents major aerodynamic challenges due to its extremely low density, approximately 1.3% of Earth’s atmospheric density, which drastically reduces lift generation and places aerial vehicles in a low-Reynolds-number flight regime. Under such conditions, conventional aerodynamic configurations become less efficient, motivating the investigation of alternative flight mechanisms capable of generating sufficient aerodynamic forces in rarefied environments. In nature, insects achieve efficient flight under similarly low Reynolds numbers by exploiting unsteady aerodynamic mechanisms, such as leading-edge vortex formation and delayed stall, which allow them to maintain lift during flapping motion [1]. These mechanisms have inspired the development of flapping-wing micro-air vehicles (MAVs), which aim to reproduce key aspects of insect flight to achieve efficient aerodynamic performance at small scales. As a result, bio-inspired flapping propulsion has emerged as a promising approach for aerial vehicles operating in unconventional environments, including planetary atmospheres [2].

To address the challenges of flight in low-density planetary atmospheres, several research efforts have explored bio-inspired flapping-wing micro-air vehicles (MAVs) as an alternative to conventional aerial platforms. These systems aim to exploit the aerodynamic mechanisms observed in insect flight, where lift generation is dominated by unsteady flow phenomena such as leading-edge vortices and wake interactions. Experimental and theoretical studies of insect aerodynamics have shown that these vortex-based mechanisms enable efficient lift generation at low Reynolds numbers, making them particularly relevant for aerial vehicles operating in rarefied environments [2]. Building on these biological principles, researchers have developed a range of flapping-wing robotic platforms designed to replicate insect-like flight mechanisms. Early experimental MAV systems demonstrated the feasibility of controlled flapping flight using lightweight actuation systems and flexible wings, providing valuable insights into the coupling between wing motion, aerodynamic forces, and flight stability [3]. Subsequent studies have expanded this research by combining computational fluid dynamics, aerodynamic modeling, and experimental testing to better understand the performance and control of flapping-wing MAVs across different Reynolds number regimes [1].

In parallel, several investigations have explored the potential of insect-inspired flapping vehicles specifically for Mars exploration. The NASA Innovative Advanced Concepts (NIAC) Marsbee concept [4] proposes a swarm of bumblebee-inspired aerial scouts capable of performing cooperative reconnaissance missions in the Martian atmosphere. In this architecture, small flapping drones would be deployed from a rover to conduct atmospheric measurements, terrain mapping, and localized exploration. Numerical studies of dynamically scaled insect-inspired wings indicate that hovering flight on Mars may be achievable when wing geometry, flapping amplitude, and frequency are appropriately adjusted to compensate for the reduced atmospheric density. More broadly, research in flapping-wing MAV design has increasingly focused on understanding the interaction between wing kinematics, structural flexibility, and aerodynamic performance. Biological studies emphasize that insect wings are not rigid structures but deform dynamically during flight, influencing aerodynamic force production and overall efficiency [2]. Consequently, both experimental and computational investigations continue to examine how wing flexibility and structural properties affect lift generation and power requirements in flapping flight systems.

Design strategies for flapping-wing MAVs typically aim to reproduce key aerodynamic and structural characteristics observed in insect flight while maintaining mechanical simplicity and manufacturability. A central aspect of these strategies is the representation of the wing structure, as aerodynamic performance in flapping flight is strongly influenced by the interaction between wing deformation, structural stiffness, and unsteady flow dynamics. Many MAV designs adopt simplified wing architectures, often consisting of rigid or membrane-based wings supported by lightweight frames. These configurations allow for relatively simple manufacturing and reduced structural mass while enabling basic aeroelastic deformation during the flapping cycle. Experimental robotic platforms using such wing structures have demonstrated that controlled flexibility can enhance aerodynamic performance by improving vortex formation and delaying flow separation during the stroke cycle [5].

Another common approach is to incorporate bio-inspired structural features derived from insect wing morphology, such as vein-like supporting elements or distributed stiffness patterns intended to approximate the mechanical behavior of natural wings. Studies in insect flight biomechanics emphasize that real insect wings exhibit complex spatial variations in stiffness and passive deformation during flight, which play a significant role in aerodynamic efficiency and force generation [2]. Consequently, several research efforts attempt to capture these effects through simplified venation patterns, flexible membranes, or parameterized stiffness models integrated into aerodynamic simulations [1].

**Figure 1:**
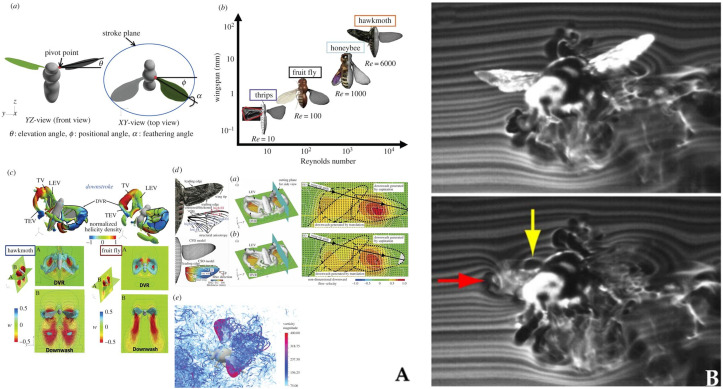
(**A**) Kinematics and prominent aerodynamic features in insect flapping flights: (a) wing morphologies of hawkmoth, honeybee, fruit fly and thrips as well as relationship of wingspan versus Reynolds number. (b) wing kinematics of a hovering fruit fly. (c) near-and far-field vortex dynamics in fruit fly and hawkmoth hovering. (d) vortex dynamics around a bumblebee flying in turbulence. (e) vortex dynamics around a bumblebee flying in turbulence [2]; (**B**) Bumblebee in sideways flights, smokelines becoming entrained in counter-rotating wing tip vortices (red arrow) and wing root vortices (yellow arrow). Frames separated by 1 ms [6].

More recently, advanced computational and experimental studies have explored the role of aeroelastic coupling in flapping-wing flight, highlighting the importance of accurately representing wing flexibility and deformation in MAV design. These investigations suggest that the interaction between wing structural properties and aerodynamic forces significantly influences lift production, power consumption, and flight stability, making wing structural design a critical component of flapping MAV performance. Figure 1 present kinematics and prominent aerodynamic features in insect flapping flights.

Despite significant progress in the development of flapping-wing MAVs, several challenges remain in translating biologically inspired flight mechanisms into practical aerial systems. One of the primary difficulties lies in accurately reproducing the structural and aeroelastic behaviour of insect wings, which strongly influences aerodynamic performance. In natural systems, insect wings exhibit complex spatial variations in stiffness and passive deformation patterns that interact with the surrounding airflow during the flapping cycle. Replicating this behaviour in engineered systems remains challenging, particularly when balancing aerodynamic fidelity with structural simplicity and manufacturability [7]. Many MAV platforms therefore rely on simplified wing representations, such as rigid wings, uniform membrane structures, or simplified venation patterns. While these configurations allow for lightweight construction and experimental testing, they often provide limited control over the distribution of stiffness and deformation across the wing surface. As a result, the resulting aeroelastic response may differ significantly from the behaviour observed in biological wings, which can influence lift generation, aerodynamic efficiency, and overall flight performance [1]. Another limitation arises from the difficulty of systematically tailoring wing mechanical properties during the design process. In many existing MAV designs, wing flexibility is determined primarily by material selection or basic geometric features rather than by a deliberate engineering of stiffness distributions across the structure. This makes it challenging to optimize the coupling between structural deformation and aerodynamic forces, particularly in the unusual aerodynamic regime associated with low-density planetary atmospheres. Furthermore, experimental validation of flapping-wing MAV concepts remains technically demanding. The interaction between wing kinematics, structural deformation, and unsteady aerodynamic forces creates a strongly coupled aeroelastic system that is difficult to model accurately and challenging to reproduce experimentally, especially under the low-pressure conditions representative of the Martian atmosphere. These challenges highlight the need for new wing design approaches that enable greater control over structural stiffness and deformation while remaining compatible with lightweight, manufacturable MAV architectures.

Bio-inspired flapping flight offers a promising alternative, as it exploits unsteady aerodynamic mechanisms, including vortex generation and wake interaction, to enhance lift under low-Reynolds-number conditions. In particular, natural flyers operate through a complex interplay between wing kinematics, structural deformation, and surrounding flow structures, enabling efficient flight in regimes where steady aerodynamics are insufficient.

Despite this potential, the fundamental mechanisms governing low-Reynolds-number flapping flight remain insufficiently understood. In particular, the coupled interaction between structural deformation and unsteady aerodynamic effects in bio-inspired flapping wings has not yet been fully characterized. Establishing these baseline aeroelastic and aerodynamic behaviors is a necessary preliminary step before extending such analyses to low-density planetary environments such as the Martian atmosphere.

## 2. Materials and Methods

### 2.1. Multi-Method Framework

To investigate the coupled aerodynamic and structural mechanisms governing flapping flight in low-Reynolds-number conditions, a multi-method framework was developed, integrating experimental observations with structural and aerodynamic modelling. The objective of this approach is to capture the interaction between wing kinematics, aeroelastic response, and unsteady flow structures, which cannot be resolved through a single modelling strategy alone.

Experimental kinematic data were obtained using high-speed imaging, enabling the extraction of key motion parameters such as flapping frequency, stroke amplitude, wing rotation, and wingtip trajectory. These measurements provide a physically grounded basis for defining representative wing motion and serve as direct inputs for both structural and aerodynamic analyses.

The structural response of the wing was investigated using finite element analysis (FEA), focusing on the identification of modal characteristics and deformation patterns under dynamic loading. Particular attention was given to the interaction between bending and torsional modes, as well as to the influence of excitation frequency relative to the natural frequencies of the structure. This enables the assessment of aeroelastic coupling mechanisms and their role in shaping the effective wing kinematics.

Aerodynamic performance was evaluated using a hierarchical modelling strategy. A low-fidelity approach based on the Unsteady Vortex Lattice Method (UVLM) was initially employed to provide rapid, preliminary insights into the unsteady aerodynamic response. However, due to the inherent limitations of inviscid formulations in capturing flow separation and vortex dynamics, higher-fidelity simulations were conducted using computational fluid dynamics (CFD). These simulations resolve viscous effects and enable the analysis of key phenomena such as leading-edge vortex formation and wake development.

The integration of these methods allows for a consistent analysis across multiple physical domains, linking experimentally observed kinematics to structural dynamics and resulting aerodynamic forces. This multi-method framework provides a comprehensive basis for identifying the governing mechanisms of flapping flight under low-density conditions.

### 2.2. Kinematic Extraction

A preliminary kinematic characterization of flapping flight was conducted based on high-speed imaging, enabling the extraction of key motion parameters relevant to subsequent analyses. These parameters were extracted for a series of high-speed images of the Apis mellifera bee, in free flight.

The observed flapping frequency, on the order of 215 Hz, together with a stroke amplitude of approximately 126°, provides a representative description of the wingbeat under high-lift conditions. In addition, the wing exhibits continuous rotation throughout the flapping cycle, indicating a strong coupling between translational and rotational motion.

**Figure 2:**
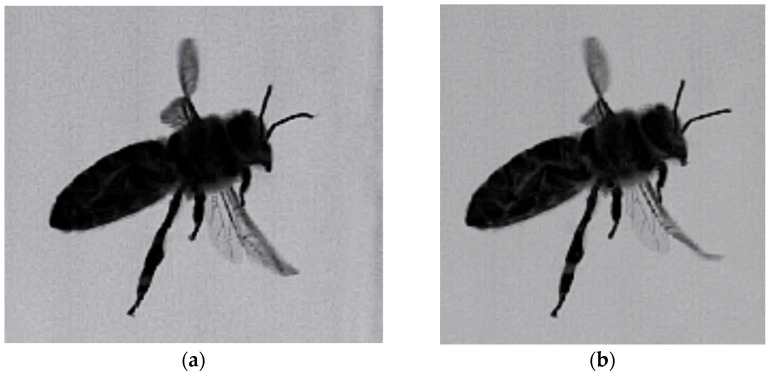
Profile view of the Apis mellifera bee in flapping flight with visible membrane deformation: (**a**,**b**) mid-upstroke consecutive stills. The deformation patterns highlight the aeroelastic response of the wing under unsteady aerodynamic loading.

**Figure 3:**
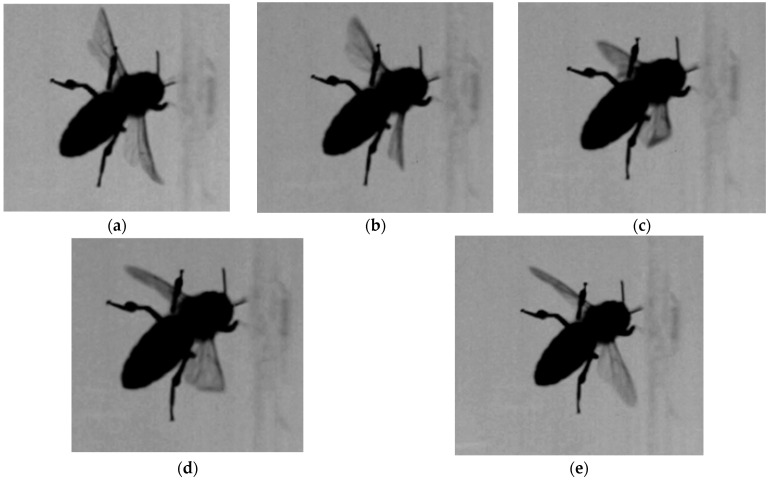
High-speed image sequence of a bee during flapping flight over a full wingbeat cycle: (**a**–**e**) successive frames capturing the transition from downstroke to upstroke. The images illustrate the large stroke amplitude, continuous wing rotation, and noticeable membrane deformation throughout the cycle, highlighting the strongly unsteady and aeroelastic nature of the motion.

These kinematic features are not treated merely as descriptors of biological flight, but as physically grounded inputs for the structural and aerodynamic modelling framework. In particular, they define the excitation conditions for the aeroelastic analysis and the prescribed motion in the aerodynamic simulations. Representative image sequence, including the observed membrane deformation during the flapping cycle, are presented in Figure 2 and Figure 3 to illustrate the characteristic wing motion and its associated structural response. Both image sequences were captured in real light, free-flight at 7500 fps.

**Figure 4:**
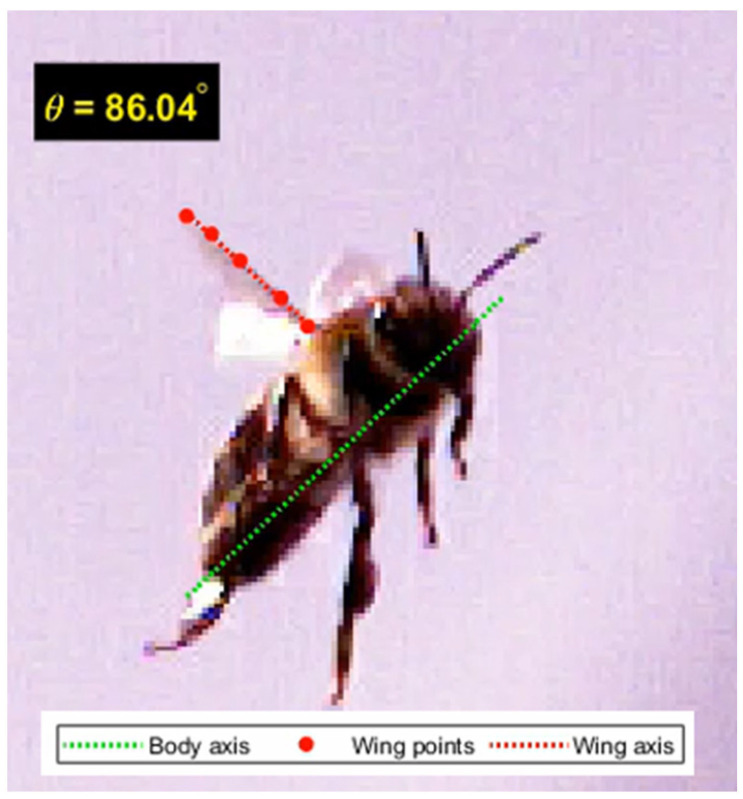
Extraction of wing kinematic parameters from high-speed imaging. The body axis (green dashed line) and wing axis (red dotted line) are defined based on tracked points along the wing (red markers), enabling the measurement of the instantaneous stroke angle (θ ≈ 86°).

To quantify the wing kinematics, key geometric parameters were extracted from high-speed image sequences of a bee in free flight. In particular, the relative orientation between the wing and the body was measured by identifying characteristic points along the wing and defining reference axes for both the body and the wing (Figure 4). This allows the estimation of the instantaneous stroke angle and provides a consistent basis for reconstructing the wing motion. The extracted angle is representative of the large-amplitude flapping regime associated with lift generation and is subsequently used to inform the prescribed kinematics in the aeroelastic and aerodynamic modelling.

The extracted kinematic parameters provide a physically grounded description of the flapping motion, capturing both the large-amplitude stroke and the continuous rotational behaviour of the wing. Together with the observed membrane deformation, these results highlight the inherently coupled nature of wing motion and structural response in flapping flight. Rather than representing purely prescribed kinematics, the motion reflects the interaction between inertial, aerodynamic, and elastic effects. As such, the derived parameters are used as representative inputs for the subsequent structural and aerodynamic analyses, ensuring consistency between experimentally observed behaviour and the modelling framework.

### 2.3. Structural Modelling (FEA)

#### 2.3.1. Bio-Inspired Wing Geometry Reconstruction

**Figure 5:**
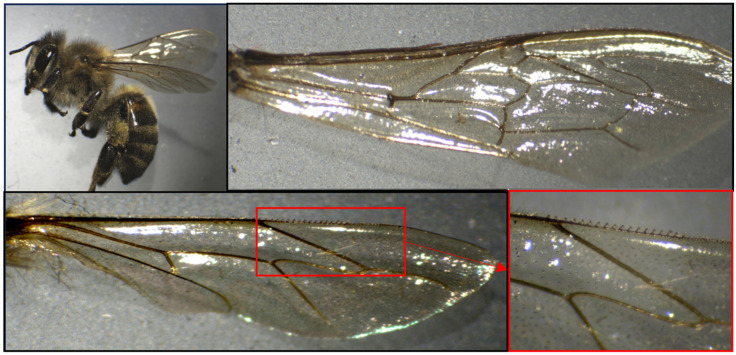
Optical microscopy images of the Apis mellifera and its forewing used for geometric reconstruction. Top left: whole specimen; top right: full wing view showing vein network and membrane structure; bottom: detailed views of the wing, including a magnified region (highlighted) illustrating vein connectivity and fine structural features along the leading edge. These observations form the basis for the bio-inspired CAD model used in the structural analysis.

To inform the structural modelling, a bio-inspired wing geometry was derived from direct observations of a bee specimen. High-resolution optical microscopy was used to capture the morphological features of the forewing, including vein topology, membrane layout, and local geometric variations. The specimen was opportunistically obtained and examined post-mortem (Figure 5), allowing detailed imaging without interference from motion or deformation associated with live flight.

The acquired images were used to reconstruct a representative wing geometry in a computer-aided design (CAD) environment. Particular attention was given to preserving the primary vein network and overall planform, which are known to play a critical role in defining the stiffness distribution and aeroelastic behaviour of the wing. While the internal microstructure of the veins could not be fully resolved, the reconstructed model captures the dominant geometric features governing the structural response.

This bio-inspired reconstruction provides a physically grounded basis for subsequent finite element analysis, enabling the investigation of deformation patterns and modal characteristics under flapping conditions. Although simplified, the approach ensures that the essential morphological traits influencing aeroelastic coupling are retained within a manufacturable and computationally tractable model.

**Figure 6:**
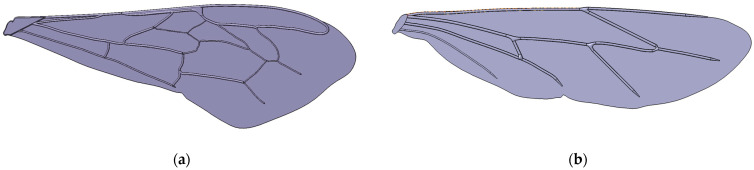
CAD models of the Apis mellifera geometrically bio-accurate (**a**) forewing and (**b**) hindwing.

The resulting models are presented in Figure 6 for both forewing and hindwing.

#### 2.3.2. Aeroelastic Response and Structural Dynamics

The structural model was geometrically scaled by a factor of ten relative to the biological wing on a simplified flat model with a membrane thickness of 0.5 mm. The material considered was Tough2000 [8] for both wing’s internal structure and membrane.

Tough2000 was selected as a simplified homogeneous structural material due to its relatively high toughness, elastic compliance, and suitability for additively manufactured thin-walled structures subjected to repeated dynamic loading. The material enables the fabrication of lightweight geometries while maintaining sufficient structural integrity during large-amplitude oscillatory motion. In the present study, the use of uniform material properties allows the dominant modal behaviour and global aeroelastic response to be investigated without introducing additional complexity associated with spatially varying stiffness distributions. However, biological insect wings exhibit highly heterogeneous structural characteristics arising from the interaction between stiff veins and compliant membrane regions, leading to strongly anisotropic mechanical behaviour. Such stiffness gradients can significantly influence modal frequencies, passive twisting, local deformation patterns, and aerodynamic performance during flapping flight. The incorporation of bio-inspired heterogeneous material distributions therefore represents an important direction for future high-fidelity aeroelastic modelling.

**Figure 7:**
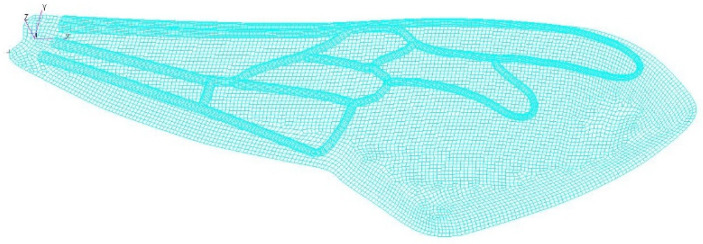
Scale model of the Apis mellifera forewing used for performing FEA.

This scaling was defined based on Reynolds number similitude, ensuring that the aerodynamic regime remains representative of the original conditions. As a consequence, the characteristic frequency of the system was reduced accordingly to preserve dynamic similarity, resulting in a lower excitation frequency for the scaled model. This approach enables the investigation of aeroelastic behaviour under conditions consistent with the underlying flow physics while maintaining numerical and structural tractability. The scaled model is presented in Figure 7. The geometric scaling factor was selected based on Reynolds number similitude between the biological wing and the scaled experimental model. The Reynolds number is defined as $Re=\frac{\rho Vl}{\mu }$ where l is the characteristic chord length and V includes both forward-flight and flapping-induced velocity components. For the biological *Apis mellifera* wing, considering a mean chord length of approximately 2 mm, a mean flapping radius of 4 mm, and a flapping frequency of 215 Hz, the estimated Reynolds number is Re≈800. For the 10× scaled model operating under low-density conditions representative of the Martian atmosphere, the resulting Reynolds number is Re≈1250. Although not identical, both configurations remain within the same low-Reynolds-number aerodynamic regime, preserving the dominant unsteady flow characteristics relevant to flapping-wing aerodynamics.

The structural behaviour of the wing was investigated using finite element analysis (FEA), with the objective of characterising its dynamic response under flapping conditions. The geometrical model was derived from the bio-inspired reconstruction and subsequently scaled by a factor of ten relative to the biological wing.

The scaling was defined based on Reynolds number similitude, ensuring that the aerodynamic regime remains representative of the original conditions. As a consequence, the increase in characteristic length was accompanied by a corresponding reduction in excitation frequency to preserve dynamic similarity.

A modal analysis was first performed to identify the dominant deformation modes of the structure. The resulting natural frequencies of the bending and torsional modes were used to define the excitation conditions for the transient simulations.

The aeroelastic response was evaluated using modal transient analysis, in which the wing was subjected to a prescribed oscillatory loading. The imposed motion consists of a combined rotation about two axes: a primary oscillation aligned with the spanwise (axial) direction and a secondary oscillation about the vertical axis. The frequency of the vertical oscillation was set to twice that of the axial motion, enabling interaction between bending and torsional deformation modes.

Two excitation cases were considered to investigate the response under different dynamic conditions. In the first case, the excitation frequency was set to 50% of the torsional natural frequency, while in the second case it was matched to the torsional mode. These conditions allow the assessment of the structural response both below and near resonance.

The transient simulations were used to extract the time evolution of bending displacement and torsional rotation, as well as their phase relationships, providing the basis for the analysis of the coupled aeroelastic behaviour.

The present structural model adopts a simplified representation of the wing as a flat plate with uniform thickness and homogeneous material properties in order to isolate the baseline aeroelastic response and maintain computational tractability. However, biological insect wings exhibit spatially varying stiffness distributions arising from the vein network and membrane heterogeneity. The inclusion of explicit venation and non-uniform structural properties would be expected to locally increase bending and torsional rigidity, particularly along the leading edge and primary load-bearing paths, resulting in modified modal frequencies and altered bending–torsion coupling behaviour. In addition, the resulting deformation patterns would likely become more spatially heterogeneous, with localized membrane deflections between veins and anisotropic stiffness effects influencing the transient response. Consequently, while the simplified model captures the dominant global dynamic behaviour, a more bio-accurate representation incorporating venation and stiffness gradients would provide improved fidelity in reproducing the passive deformation mechanisms observed in biological flapping wings.

### 2.4. Aerodynamic Modelling

#### 2.4.1. Low-Fidelity Aerodynamic Modelling (UVLM)

The aerodynamic response of the flapping wing was initially evaluated using the Unsteady Vortex Lattice Method (UVLM) implemented within the Ptera Python 3.2.0 framework. The simulations were performed for a prescribed flapping motion at a frequency of 155 Hz and a stroke amplitude of 58°, representative of hovering conditions.

UVLM is a computationally efficient, potential-flow formulation in which the lifting surface and its wake are represented by a distribution of discrete vortex rings. As an inviscid method, it neglects viscous effects and therefore cannot capture boundary layer development, flow separation, or stall. In particular, key mechanisms relevant to flapping flight, such as leading-edge vortex (LEV) formation, are not inherently resolved and require additional modelling assumptions. Furthermore, the wing is represented as a zero-thickness lifting surface defined by its mean camber, which limits the ability of the model to reproduce three-dimensional flow features associated with finite thickness and complex geometry.

**Figure 8:**
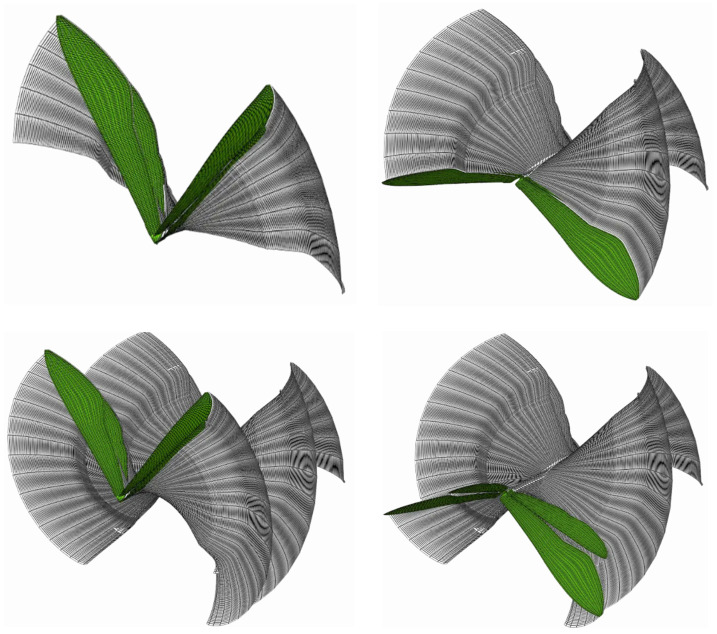
Selected frames from the UVLM simulation of the flapping wing over one cycle. The sequence illustrates the evolution of the wake structure and the distribution of shed vortices generated by the prescribed wing motion.

The simulation was conducted over two complete flapping cycles. The resulting aerodynamic loads exhibit pronounced numerical instabilities, with predicted forces and moments reaching non-physical magnitudes. For instance, the computed lift force exceeds several hundred newtons, and the pitching moment reaches unrealistically large values for a micro-scale wing. These responses do not correspond to physical aerodynamic behaviour but arise from numerical singularities inherent to the UVLM formulation under highly unsteady motion. A sequence of stills from the resulting animation are presented in Figure 8.

In particular, during stroke reversal, previously shed wake vortices are convected in close proximity to the lifting surface. As these vortices approach the collocation points of the lattice, the induced velocities can become unbounded in the absence of sufficient vortex core regularisation. This leads to a breakdown in the numerical solution and manifests as spurious spikes in the computed aerodynamic loads.

#### 2.4.2. Aerodynamic Modelling (CFD)

The study considers a two-dimensional bee wing profile with a chord length of *c* = 45.25 mm, representing both the forewing and hindwing, scaled by a factor of 10. The 2D profile is extracted from a bio-inspired wing geometry at the spanwise location where the chord length reaches its maximum value.

**Figure 9:**
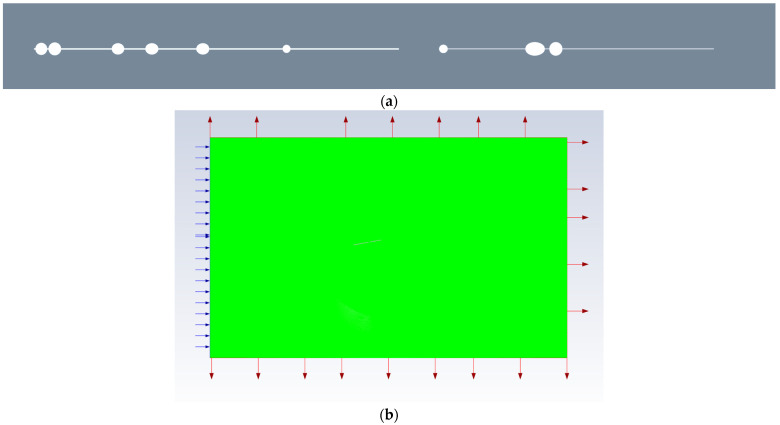
(**a**) Wing geometry; (**b**) Computational domain for the numerical simulation.

Numerical simulations were performed under laminar flow conditions using the commercial CFD solver ANSYS Fluent (version R24.2). An incompressible formulation with second-order spatial discretization was employed. The computational domain is illustrated in Figure 9, which also shows the wing profiles.

To capture the unsteady aerodynamics associated with the flapping motion, the mesh was generated in ANSYS Meshing using a dynamic mesh approach. Local remeshing and smoothing methods were employed to handle mesh deformation associated with wing motion. The mesh was generated using predominantly quadrilateral elements, with structured refinement near the wing surface to improve boundary layer resolution. A total of 15 inflation layers were used, with a first layer thickness of 2.5 × 10^−8^ m and a growth rate of 1.3.

While the present study primarily focuses on the identification of the dominant unsteady aerodynamic mechanisms associated with flapping motion, additional quantitative aerodynamic metrics would provide a more comprehensive characterization of the wing performance. Parameters such as instantaneous and cycle-averaged lift and drag coefficients, aerodynamic power consumption, vortex circulation strength, and propulsive efficiency could further quantify the aerodynamic behaviour and enable direct comparison between different wing configurations and operating conditions. Due to the simplified two-dimensional rigid-wing formulation and the exploratory nature of the present analysis, the current work emphasizes the interpretation of the underlying flow physics and aeroelastic response. The incorporation of additional quantitative aerodynamic measures within fully coupled three-dimensional simulations will therefore be considered in future work.

The flapping motion was modelled as harmonic motion representative of hovering flight [9]. Flapping angle rate ($\dot{\varphi }$) is described as:

(1) $$\dot{\varphi }=\Phi \pi fcos(2\pi ft),$$

with Φ as the amplitude, *f* is the flapping frequency, 155 Hz. The pitching angle rate ($\dot{\alpha }$) is given as:

(2) $$\dot{\alpha }=0.5\dot{\dot{\alpha_{0}}\left\{1-cos\left[\frac{2\pi (\tau -\tau_{D})}{\Delta \tau_{r}}\right]\right\}},$$

where $\tau_{D}$ is start of wing rotation during downstroke, $\Delta \tau_{r}$ the duration of wing rotation and $\dot{\alpha_{0}}$ is described as:

(3) $${\dot{\alpha }}_{0}=\frac{\pi -\alpha_{U}-\alpha_{D}}{\Delta \tau_{r}\times T}$$

During upstroke, the sign of ${\dot{\alpha }}_{0}$ is reversed, while wing rotation start is $\tau_{U}$. Both $\tau_{D}/\tau_{U}$ and ${\Delta \tau }_{r}$ are non-dimensionalised with $\tau =\frac{t}{T}$ which represents non-dimensional time. Translation velocity in the X and Y directions is given as:

(4) $$\dot{X}=\dot{\varphi }\cos(\beta )\;\dot{Y}=\dot{\varphi }\sin(\beta )$$

where the stroke angle is fixed, *β* = 12°. The angles of attack at mid-upstroke and mid-downstroke are *α_U_* = 21° and *α_D_* = 27°, respectively. The pitching axis is located at 0.3*c* from the leading edge.

For this case, three-dimensional effects were neglected, and the wings were modelled as rigid surfaces, without accounting for flexibility or twisting. Spatial discretization employed a second-order upwind scheme, while temporal terms were discretised based on a second-order implicit method. A coupled pressure-velocity solver was used for better accuracy. Boundary conditions consisted of pressure outlets on all outer domain surfaces and a no-slip wall condition on the wing surfaces. The flow was treated as unsteady and incompressible.

The present aerodynamic model adopts a two-dimensional rigid wing representation in order to isolate the dominant unsteady flow mechanisms while maintaining computational tractability. However, real insect wings exhibit strongly three-dimensional and aeroelastic behaviour, including spanwise flow, wingtip vortex formation, passive twisting, and membrane deformation during the flapping cycle. These effects can significantly influence vortex stability, aerodynamic damping, and force generation. In addition, the rigid-wing assumption neglects the feedback between aerodynamic loading and structural deformation, which is known to contribute to the aerodynamic efficiency of biological flapping wings. Consequently, the present CFD results should be interpreted as a preliminary characterization of the unsteady aerodynamic response rather than a fully resolved representation of insect flight aerodynamics. Future work will therefore focus on extending the model toward three-dimensional flexible wing simulations with fully coupled fluid–structure interaction.

## 3. Results

### 3.1. Kinematic Features of Flapping Motion

The kinematic analysis of the flapping motion was performed based on high-speed image sequences, allowing the extraction of time-resolved parameters over a full wingbeat cycle. The observed flapping frequency, on the order of 215 Hz, together with a stroke amplitude of approximately 126°, provides a representative description of the wingbeat under high-lift conditions. In addition, the wing exhibits continuous rotation throughout the flapping cycle, indicating a coupled translational and rotational motion.

**Figure 10:**
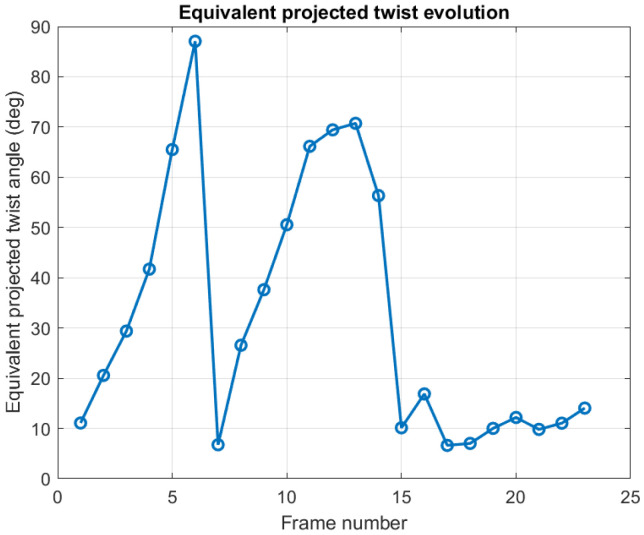
Temporal evolution of the equivalent projected twist angle over the flapping cycle, showing significant variation in wing rotation across successive frames.

The temporal evolution of the equivalent projected twist angle is presented in Figure 10. The twist angle varies significantly throughout the cycle, with values ranging from approximately 10° to nearly 90°. A pronounced peak is observed in the early portion of the sequence, followed by a rapid decrease and a subsequent secondary increase, indicating a non-uniform distribution of rotational motion across the wingbeat.

**Figure 11:**
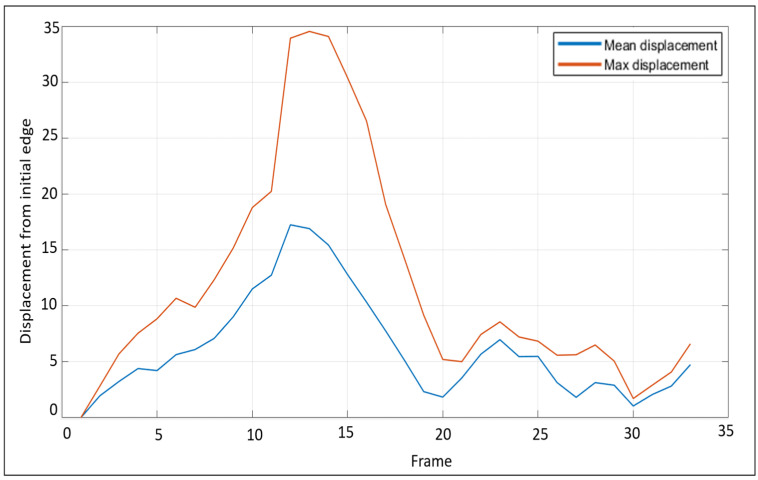
Leading-edge displacement during the flapping cycle as a function of frame number. Mean and maximum displacement values are shown, illustrating the temporal evolution of wing motion along the leading edge.

The displacement of the leading edge relative to its initial position is shown in Figure 11, where both mean and maximum values are reported. The leading-edge motion exhibits a gradual increase during the initial phase of the cycle, reaching peak values in the mid-cycle frames, followed by a decrease toward the end of the sequence. The maximum displacement consistently exceeds the mean displacement, reflecting spatial variations along the leading edge.

Together, these results provide a quantitative description of the flapping kinematics, capturing the temporal variation in wing rotation and deformation over the cycle.

### 3.2. Aeroelastic Response

The structural behaviour of the wing was investigated using finite element analysis, with the objective of characterising the dynamic response and modal interactions under flapping conditions. The geometrical model was derived from the bio-inspired reconstruction and subsequently scaled by a factor of ten based on Reynolds number similitude, ensuring that the aerodynamic regime remains representative while enabling tractable structural analysis. To preserve similarity, the increase in characteristic length was accompanied by a corresponding reduction in excitation frequency.

A preliminary modal analysis was performed to identify the dominant deformation modes of the structure. The first bending and torsional natural frequencies were determined as $f_{b}=5.96\;\mathrm{Hz}$ and $f_{t}=9.03\;\mathrm{Hz}$, respectively. These modes define the dynamic characteristics of the wing and are used to establish the excitation conditions for the transient response analysis.

The aeroelastic response was evaluated using modal transient simulations, in which the wing is subjected to a prescribed oscillatory loading combining rotations about two axes: a primary oscillation aligned with the spanwise (axial) direction and a secondary oscillation about the vertical axis. The vertical oscillation is imposed at twice the frequency of the axial motion, enabling interaction between bending and torsional modes.

**Figure 12:**
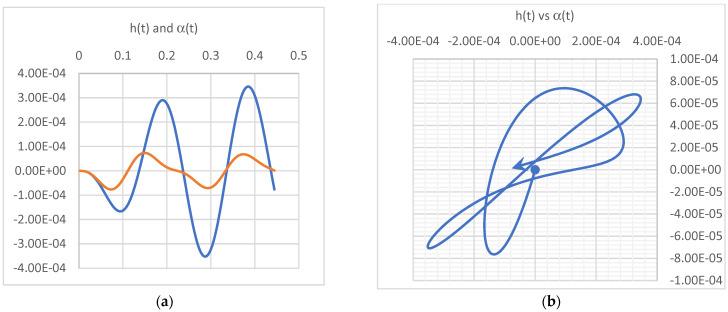

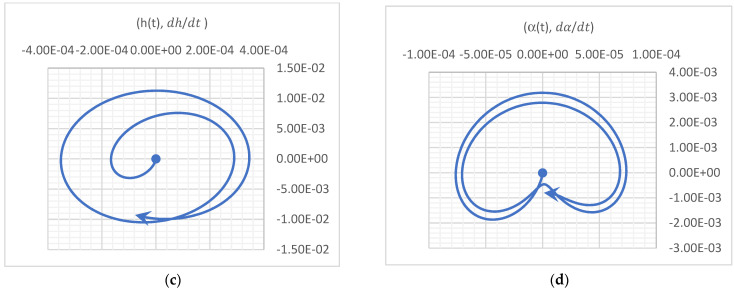
Modal transient response, and phase diagram for excitation frequency 4.5 Hz: (**a**) h(t), α(t), (**b**) h(t) vs. α(t), (**c**) Phase diagram (h(t), $\frac{dh}{dt}$) and (**d**) Phase diagram (α(t), $\frac{d\alpha }{dt}$).

Two excitation cases were considered. In the first case, the excitation frequency was set to f=4.5 Hz, corresponding to approximately 50% of the torsional natural frequency. The resulting transient response is shown in Figure 12, including the time evolution of the bending displacement h(t), torsional rotation α(t), and the associated phase-space representations.

For this excitation condition, the response exhibits coupled oscillatory behaviour between bending and torsion, with a phase shift between the modal amplitudes. The corresponding phase diagrams indicate a bounded trajectory, reflecting a quasi-periodic structural response.

**Figure 13:**
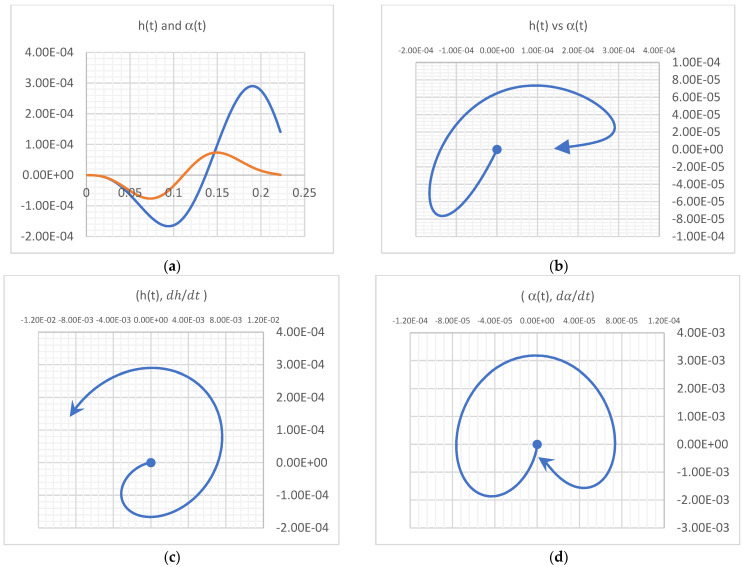
Modal transient response, and phase diagram for excitation frequency 9 Hz: (**a**) h(t), α(t), (**b**) h(t) vs. α(t), (**c**) Phase diagram (h(t), $\frac{dh}{dt}$) and (**d**) Phase diagram (α(t), $\frac{d\alpha }{dt}$).

In the second case, the excitation frequency was set equal to the torsional natural frequency, f=9 Hz. The corresponding transient response is presented in Figure 13, again showing the modal amplitudes and phase-space representations.

Under this condition, a stronger interaction between torsional and bending modes is observed. The bending amplitude increases over time, and the phase-space trajectories exhibit an expanding pattern, indicating a growth in the structural response. The phase relationship between torsion and bending reflects a transfer of energy between modes.

The observed near-resonant behaviour also has important practical implications for the design of flapping-wing systems. Operating close to structural resonance can increase deformation amplitudes and enhance passive wing motion, potentially improving aerodynamic effectiveness while reducing actuation requirements. However, near-resonant operation may also increase sensitivity to excessive deformation, dynamic instability, material fatigue, and reduced controllability, particularly in lightweight flexible wing configurations. Consequently, practical flapping-wing designs require a balance between structural compliance and dynamic stability, where resonance tuning is carefully controlled to exploit beneficial passive deformation effects without compromising structural integrity or flight robustness.

Overall, the results demonstrate a pronounced dependence of the aeroelastic response on the excitation frequency relative to the natural modes, with distinct dynamic behaviour observed for sub-resonant and resonant conditions.

### 3.3. Aerodynamic Response

#### 3.3.1. UVLM

The aerodynamic loads predicted by the UVLM simulations were analysed over two flapping cycles in terms of forces, moments, and their corresponding coefficients.

**Figure 14:**
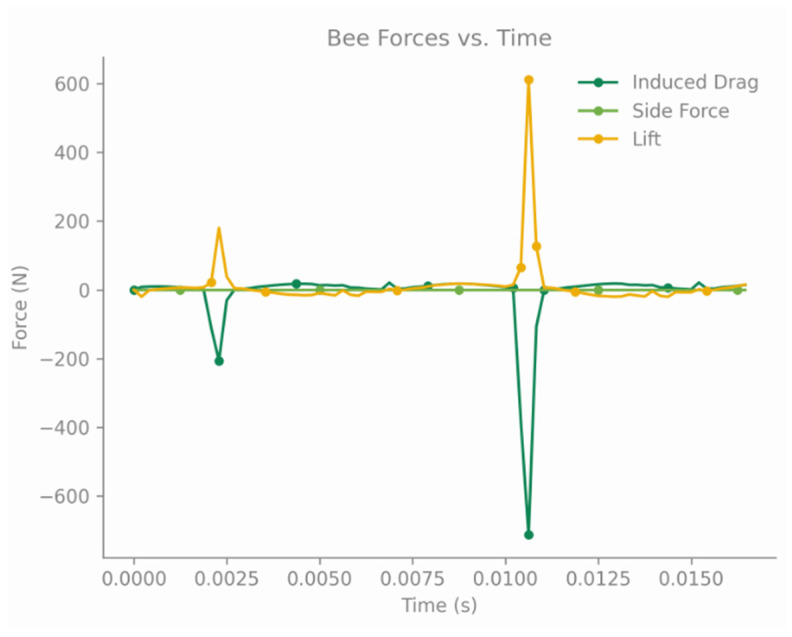
Aerodynamic force components predicted by UVLM as a function of time, including lift, induced drag, and side force over the flapping cycle.

The time evolution of the aerodynamic force components is presented in Figure 14, including lift, induced drag, and side force. The results show strong temporal variations throughout the flapping cycle, with pronounced peaks occurring at specific time instances. In particular, the lift force exhibits sharp transient increases, while the induced drag shows corresponding large negative excursions. The side force remains comparatively small in magnitude over the cycle.

**Figure 15:**
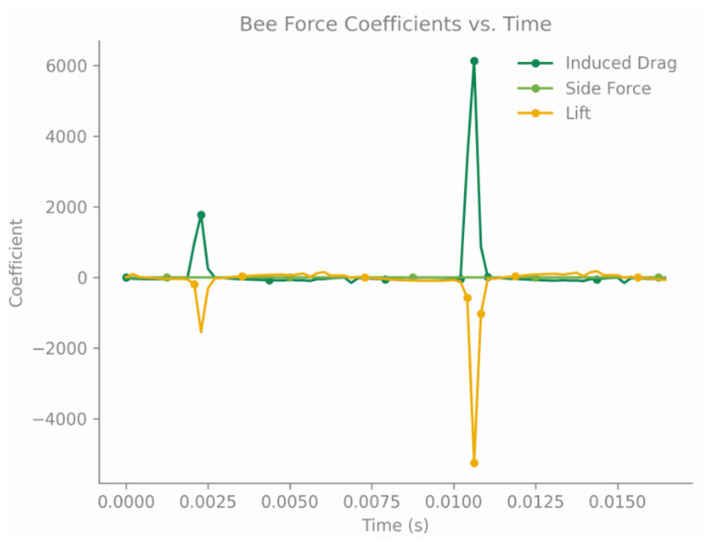
Aerodynamic force coefficients as a function of time, showing the temporal evolution of lift, induced drag, and side force in non-dimensional form.

The corresponding non-dimensional force coefficients are shown in Figure 15. Similar trends are observed, with the lift and induced drag coefficients exhibiting large fluctuations, including abrupt spikes of significant magnitude. These variations occur over short time intervals and are not uniformly distributed across the cycle.

**Figure 16:**
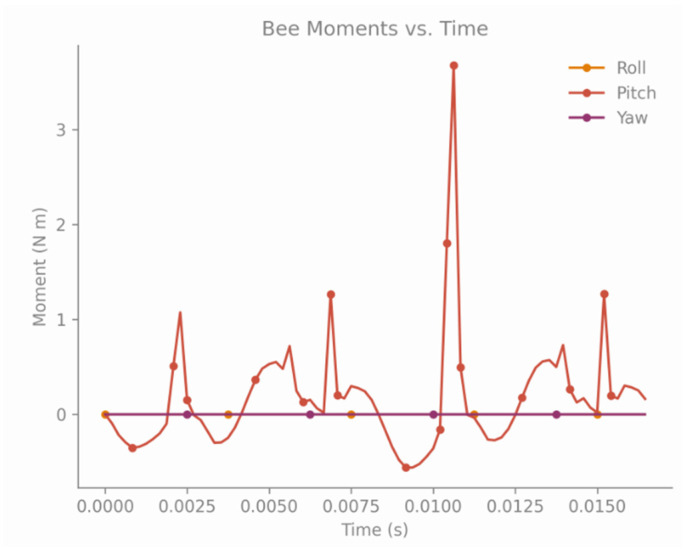
Aerodynamic moments about the principal axes (roll, pitch, yaw) as a function of time over the flapping cycle.

The aerodynamic moments about the principal axes are presented in Figure 16, showing roll, pitch, and yaw components. The pitch moment dominates the response, with significantly larger amplitudes compared to roll and yaw. The time history reveals sharp peaks and rapid variations, particularly in the pitching component.

**Figure 17:**
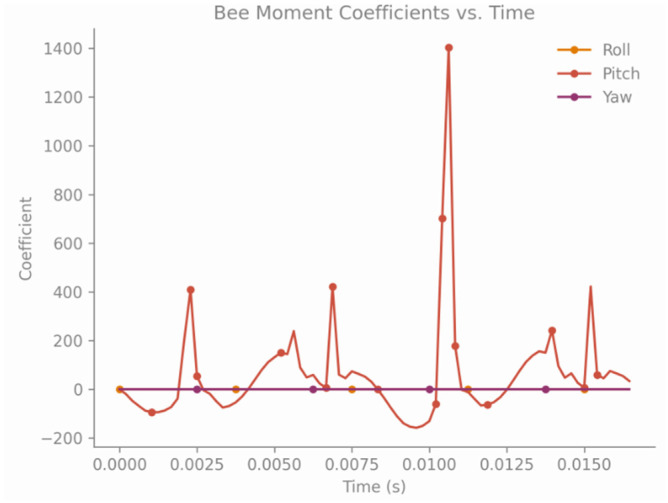
Aerodynamic moment coefficients as a function of time, showing the temporal variation in roll, pitch, and yaw contributions.

The corresponding moment coefficients are shown in Figure 17, where similar behaviour is observed in non-dimensional form. The pitch coefficient exhibits pronounced spikes, while the roll and yaw coefficients remain close to zero throughout the simulation.

Overall, the UVLM results show highly unsteady aerodynamic loads characterized by large-amplitude fluctuations and short-duration peaks in both force and moment responses.

#### 3.3.2. CFD Results

The aerodynamic force generated by the wing is strongly governed by the temporal evolution of the vortex structures. In particular, wake capture, wing–wing interaction, vortex formation and shedding play a central role in modulating the instantaneous lift and drag during the flapping cycle.

**Figure 18:**
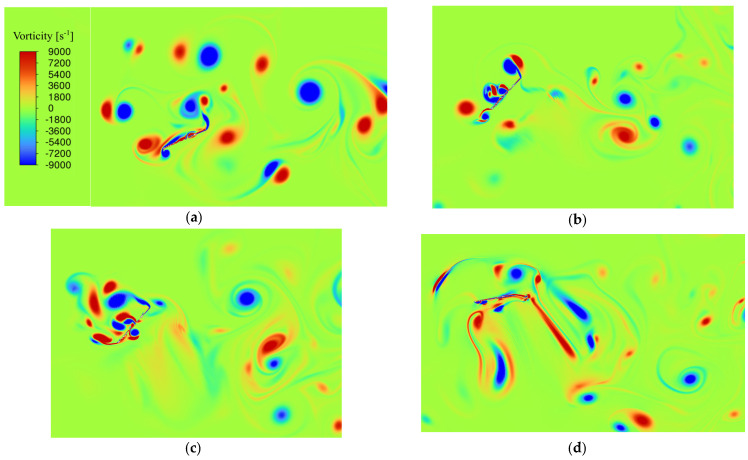
Flow field snapshots (vorticity distribution) around a 2D bee wing profile in hovering flight: (**a**) t/T = 7.75; (**b**) t/T = 12.4; (**c**) t/T = 15.5; (**d**) t/T = 18.6.

Figure 18 presents the instantaneous flow field of the bee wing at a fixed stroke angle of $\beta =12^{\circ }$. At t/T=7.75, the flow field is dominated by strong vortices structures. A leading-edge vortex forms on the fore wing and remains attached over a significant portion of the chord, while multiple vortices of opposite sign are shed into the wake. In the vicinity of the hindwing, these structures interact and intensify, leading to a highly rotational flow region.

At t/T=12.4, the vorticity field becomes less organized. The leading-edge vortex on the forewing weakens and begins to lose coherence, while the wake behind the wing contains smaller, more dispersed vortical structures. The interaction between the fore and hind wings is still present but less pronounced, as the vortices convect downstream and diffuse. As the cycle progresses to t/T=15.5, the flow field is characterized by scattered and weakened vorticity, with no dominant vortex structures present on either wing. The wake becomes largely diffused, indicating that vortex-induced lift is significantly reduced and that aerodynamic interaction between the wings is minimal.

At t/T=18.6, the vorticity field shows the early development of new flow structures. Although the overall vorticity magnitude remains relatively low, emerging shear layers indicate the onset of vortex formation associated with the next stroke. Thus, even though the instantaneous aerodynamic forces are small, the flow is not inactive but is instead evolving toward the next cycle.

**Figure 19:**
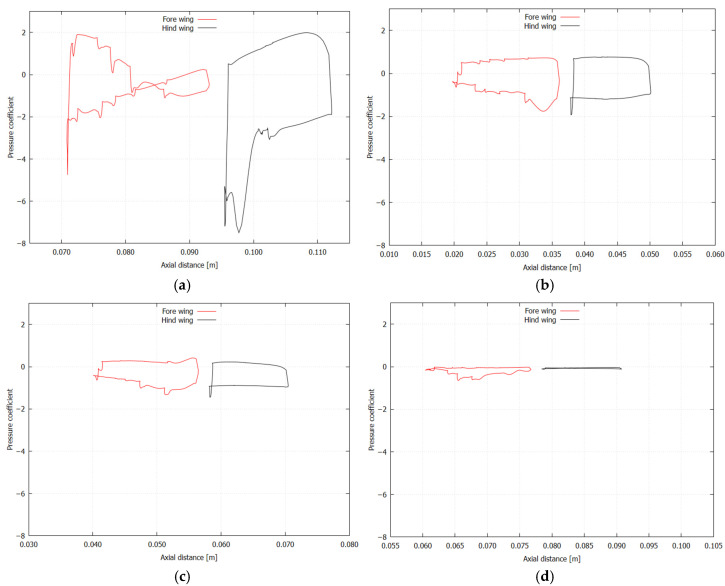
Pressure coefficient distribution around a 2D bee wing profile in hovering flight: (**a**) t/T = 7.75; (**b**) t/T = 12.4; (**c**) t/T = 15.5; (**d**) t/T = 18.6.

The pressure coefficient distributions presented in Figure 19 further illustrate the highly unsteady aerodynamic behavior of the configuration. At t/T=7.75 large fluctuations in Cp are observed along both the fore and hind wings. The fore wing exhibits significant differences between the upper and lower surfaces, consistent with the presence of a strong leading-edge vortex and flow separation. The hind wing shows even more pronounced negative Cp values on its lower surface, indicating strong vortex-induced suction associated with the interaction with vortical structures shed by the forewing.

As the phase advances to t/T=12.4, the pressure distributions become smoother for both wings, indicating a partial weakening of unsteady effects. The pressure difference between the upper and lower surfaces decreases, reflecting a reduction in aerodynamic loading. This trend corresponds to the loss of coherence in the vortical structures, which convect downstream and dissipate. Although the hind wing still experiences some influence from the upstream wake, the interaction is less intense, resulting in reduced lift compared to the earlier phase.

At t/T=15.5, the pressure distributions show further stabilization, with relatively small variations in Cp along both surfaces of each wing. The reduced pressure difference indicates a continued decline in lift generation, consistent with the absence of strong, coherent vortices in the flow field.

Finally, at t/T=18.6, the pressure coefficient remains close to zero over most of the chord for both wings, indicating a phase of minimal aerodynamic loading. Although the pressure field appears weak, this phase corresponds to the early stages of flow reorganization, during which new vortical structures begin to form in preparation for the next cycle.

Overall, the results demonstrate that the aerodynamic performance is highly phase-dependent, with peak lift occurring during periods of strong vortex formation and wing–wing interaction, and reduced performance associated with vortex breakdown and flow reorganization.

## 4. Discussion

### 4.1. Coupled Aeroelastic–Aerodynamic Behaviour

The results highlight that flapping-wing performance is governed by a strong coupling between structural dynamics and unsteady aerodynamic phenomena. The kinematic analysis demonstrates large stroke amplitudes and continuous wing rotation, while the structural simulations reveal a pronounced interaction between bending and torsional modes. In particular, the phase relationship between these modes varies with excitation frequency, indicating that the effective wing motion is not purely prescribed but emerges from the interaction between inertial, elastic, and aerodynamic effects. This coupling plays a central role in shaping the instantaneous wing configuration during the flapping cycle. The deformation of the wing modifies the local angle of attack and camber distribution, which in turn affects the aerodynamic loading. The results therefore suggest that aeroelastic deformation is an intrinsic component of lift generation rather than a secondary effect.

### 4.2. Role of Resonance and Modal Interaction

The structural analysis shows that the response of the wing is highly sensitive to the excitation frequency relative to the natural modes. Near the torsional natural frequency, the interaction between bending and torsion becomes significantly amplified, leading to increased deformation and evolving phase relationships.

This behaviour is indicative of a resonance-driven regime, in which energy transfer between modes becomes more pronounced. The observed growth in bending amplitude under near-resonant excitation suggests that the structural response can be enhanced through appropriate tuning of the excitation frequency. At the same time, the results also indicate the onset of dynamic instability when the response becomes unbounded, highlighting the need for a balance between amplification and stability.

### 4.3. Assessment of Aerodynamic Modelling Approaches

The comparison between UVLM and CFD results provides insight into the capabilities and limitations of different aerodynamic modelling strategies for flapping flight.

The UVLM simulations reproduce the general unsteady nature of the aerodynamic loads but exhibit pronounced numerical instabilities, manifested as large, non-physical spikes in both forces and moments. These instabilities are associated with the interaction between the wing and its wake, particularly during stroke reversal, where the assumptions of the inviscid formulation break down. As a result, UVLM is shown to be insufficient for capturing the complex flow physics associated with highly unsteady flapping motion.

In contrast, the CFD simulations resolve the viscous flow field and provide a more physically consistent representation of the aerodynamic response. The results show the formation and evolution of coherent vortical structures, including leading-edge vortices and wake shedding, which are characteristic of low-Reynolds-number flapping flight. These structures persist over a significant portion of the flapping cycle and contribute to sustained lift generation.

### 4.4. Implications for Low-Density Flight

The combined results indicate that efficient flapping flight in low-density environments relies on the interaction between aeroelastic deformation and unsteady vortex dynamics. The large stroke amplitudes and high flapping frequencies observed in the kinematic analysis are consistent with the need to enhance unsteady lift mechanisms under low Reynolds number conditions.

Furthermore, the sensitivity of the structural response to resonance suggests that operating near specific modal frequencies can enhance aerodynamic performance. However, the transition to unstable behaviour under certain conditions highlights the importance of controlling the structural response to avoid detrimental effects.

### 4.5. Limitations and Future Work

Several limitations of the present study should be noted. The CFD analysis is based on a two-dimensional wing model, which does not capture three-dimensional effects such as spanwise flow and tip vortices. Additionally, the structural and aerodynamic analyses were performed in a sequential manner rather than within a fully coupled fluid–structure interaction framework. In this study, the term “structural coupling” refers specifically to the coupled bending–torsion dynamic interaction observed within the structural response of the wing. A fully coupled aeroelastic FSI implementation was beyond the exploratory scope of the present work due to the substantial computational complexity associated with transient low-Reynolds-number flapping flows and large structural deformations.

The aeroelastic behaviour observed in the present study is consistent with previous investigations of insect-inspired flapping wings, which report strong coupling between bending deformation, torsional motion, and unsteady aerodynamic loading under low-Reynolds-number conditions. Similar studies have shown that passive deformation and modal interactions can significantly influence vortex formation, lift generation, and energetic efficiency during flapping flight [10]. The present results support these observations by demonstrating frequency-sensitive structural response and coupled oscillatory behaviour near the torsional natural frequency, even within a simplified structural representation.

The aerodynamic simulations likewise exhibit qualitative features commonly reported in the literature on flapping-wing aerodynamics, including wake development, vortex-dominated flow structures, and strong transient effects associated with stroke reversal [11]. At the same time, the limitations identified in the present work, particularly the use of a two-dimensional rigid-wing formulation and the absence of fully coupled fluid–structure interaction, are consistent with challenges recognized in previous numerical studies of insect flight. Consequently, the present framework should be interpreted as a preliminary step toward more comprehensive bio-inspired flapping-wing models capable of reproducing the combined aerodynamic and aeroelastic mechanisms observed in natural flyers. Overall, the present work demonstrates that bio-inspired flapping wings operating in low-Reynolds-number conditions exhibit strong coupling between structural dynamics and unsteady aerodynamic phenomena. The combined experimental–numerical framework enabled the identification of representative flapping kinematics, modal interactions, and vortex-dominated flow behaviour relevant to low-density flight environments. The results provide a preliminary physics-based foundation for the future development of more advanced flapping-wing aeroelastic models incorporating fully coupled fluid–structure interaction and biologically representative wing structures.

The present findings also provide several preliminary implications for the design of bio-inspired flapping-wing systems operating under low-Reynolds-number conditions. In particular, the observed sensitivity of the structural response to excitation frequency suggests that resonance tuning and modal coupling may play an important role in maximizing flapping efficiency while limiting excessive structural deformation. The results further indicate that material selection, wing stiffness distribution, and passive deformation characteristics can significantly influence the resulting dynamic and aerodynamic behaviour. From an aerodynamic perspective, the simulations highlight the importance of accurately resolving unsteady vortex structures when evaluating flapping-wing performance in low-density environments. Although the present framework remains simplified, these observations provide useful guidance for the future design of lightweight flapping-wing aerial systems incorporating controlled structural compliance and bio-inspired wing architectures.

Future work should focus on fully coupled fluid–structure interaction simulations, as well as experimental validation under controlled conditions representative of low-density environments. Extending the analysis to three-dimensional wing geometries will also be essential for capturing the complete aerodynamic behaviour of flapping wings.

## 5. Conclusions

This study investigated the structural dynamic response and unsteady aerodynamic behaviour of a bio-inspired flapping wing under low-Reynolds-number operating conditions relevant to low-density flight regimes. An integrated approach combining kinematic analysis, structural modelling, and aerodynamic simulations was employed to investigate the governing mechanisms associated with flapping-wing motion.

The wing geometry was derived from an actual bee wing, while the aerodynamic model was constructed from a two-dimensional sectional profile extracted from the reconstructed biological wing, ensuring morphological consistency between the structural and aerodynamic representations.

The results indicate that the simplified wing model exhibits coupled bending and torsional dynamic behaviour, with the structural response strongly influenced by the excitation frequency relative to the natural modes. Near-resonant conditions produced amplified deformation and evolving phase relationships, highlighting the sensitivity of the structural response to modal interactions under prescribed flapping excitation.

From an aerodynamic perspective, the comparison between modelling approaches highlights the limitations of low-fidelity inviscid methods in capturing highly unsteady flapping motion, while CFD simulations resolve vortex-dominated flow structures associated with lift generation under low-Reynolds-number conditions.

Overall, the findings suggest that the interaction between structural dynamics and unsteady aerodynamic mechanisms plays an important role in flapping-wing behaviour. The present work provides a preliminary physics-based framework for the future development of bio-inspired flapping-wing systems incorporating controlled structural compliance and unsteady aerodynamic effects.

The study also highlights several important limitations associated with the present simplified framework, including the use of homogeneous material properties, a two-dimensional rigid-wing aerodynamic model, and the absence of fully coupled fluid–structure interaction. Future work will therefore focus on incorporating spatially varying stiffness distributions, aerodynamic and structural damping, and fully coupled three-dimensional aeroelastic simulations under atmospheric conditions representative of Martian flight environments.

## Data Availability

All generated data are contained in the article or can be made available upon request.
